# Using a Consumer Wearable Activity Monitoring Device to Study Physical Activity and Sleep Among Adolescents in Project Viva: Cohort Study

**DOI:** 10.2196/59159

**Published:** 2025-02-04

**Authors:** Yutong Zhang, Nicole Bornkamp, Marie-France Hivert, Emily Oken, Peter James

**Affiliations:** 1 Department of Population Medicine, Harvard Pilgrim Health Care Institute, Boston, MA, United States; 2 Diabetes Unit, Massachusetts General Hospital, Harvard Medical School, Boston, MA, United States; 3 Harvard Medical School, Harvard University, Cambridge, MA, United States; 4 Department of Nutrition, Harvard T.H. Chan School of Public Health, Harvard University, Boston, MA, United States; 5 Department of Environmental Health, Harvard T.H. Chan School of Public Health, Harvard University, Boston, MA, United States; 6 Division of Environmental and Occupational Health, Department of Public Health Sciences, University of California, Davis School of Medicine, Davis, CA, United States

**Keywords:** wearable device, Fitbit, physical activity, sleep, adolescents, behavior risk, mobile phone

## Abstract

**Background:**

The increasing prevalence of physical inactivity and insufficient sleep in adolescents likely contribute to worsening cardiometabolic and mental health. However, obtaining accurate behavioral measures is a challenge. Consumer wearable devices offer a user-friendly method to assess physical activity and sleep.

**Objective:**

This study aimed to describe the process and the preliminary results of physical activity and sleep collected using a consumer wearable Fitbit device in an adolescent cohort.

**Methods:**

We provided Fitbit Charge 2 or Charge 3 wrist-worn activity monitors to adolescent participants in Project Viva, a Boston, Massachusetts area cohort, from 2017 to 2022. We invited participants to wear the devices for ≥7 days for 24 hours a day to measure their physical activity, heart rate, and sleep, and allowed them to keep the device as a participation incentive.

**Results:**

We collected over 7 million minutes of physical activity, heart rate, and sleep data from 677 participants, 53% (356/677) of whom were female. The mean (SD) age of participants was 17.7 (0.7) years. Among the 677 participants, 65% (n=439) were non-Hispanic White, 14% (n=947) were non-Hispanic Black, 10% (n=69) were Hispanic, 3.2% (n=22) were non-Hispanic Asian, and 7.8% (n=53) belonged to other races. Participants demonstrated a high adherence to the research protocol, with the mean (SD) wear duration of 7.5 (1.1) days, and 90% of participants (612/677) had 5 or more days wearing the device for >600 minutes/day. The mean (SD) number of steps was 8883 (3455) steps/day and the mean (SD) awake sedentary time was 564 (138) minutes/day. Male participants were more often engaged in very active (27 minutes/day) and moderately active physical activity (29 minutes/day) compared with female participants (15 and 17 minutes/day, respectively). Over 87% (588/677) of participants had sleep data available for 5 or more days, among whom the average nightly sleep duration was 7.9 (SD 0.9) hours.

**Conclusions:**

This study demonstrated the feasibility of using consumer wearable devices to measure physical activity and sleep in a cohort of US adolescents. The high compliance rates provide valuable insights into adolescent behavior patterns and their influence on chronic disease development and mental health outcomes.

## Introduction

The prevalence of physical inactivity and insufficient sleep in adolescents has become a major health concern worldwide. In the United States, approximately 76% of children aged 6‐17 years engage in less than the recommended 60 minutes of moderate-to-vigorous physical activity per day [1]. Similarly, almost a quarter of US children aged 6‐17 years have less than the recommended 8 hours of sleep per day [2]. Adolescence is a vulnerable period for the development of both psychiatric and chronic medical illnesses [3]. A lack of moderate-to-vigorous physical activity is linked to a higher risk of excessive calorie intake, obesity [4,5], and cardiometabolic diseases [6-8]; higher risk of depression and anxiety [9-11]; and lower cognitive and school performance [12,13]. In addition, insufficient sleep has been associated with unhealthy dietary behavior [14,15], being overweight [14,16,17], poor school performance, and depression [18-20]. Given the health concerns related to physical inactivity and insufficient sleep among adolescents, a better understanding of these health behaviors through accurate, large-scale data among representative populations is crucial.

Consumer-based wearable devices such as Fitbit (Google) have become popular for objectively measuring physical activity and sleep due to the advancements in microtechnology, wireless communication, battery capacity, and multidimension measurements. In addition, Fitbit devices use Bluetooth for easy data transfer to Fitbit servers through a smartphone or tablet, allowing for synchronization of real-time data. This helps avoid the burden of mailing devices back to researchers and potential data loss that may occur with actigraphs meant solely for research. Fitbits have been validated for collecting real-time behavior data on free-living subjects [21-25]. Although Fitbits have been used to collect real-time behavior data, researchers have not described real-world methods to operationalize consumer-based wearable devices to collect health behavior data in adolescent cohorts. Detailing the operational methods for these devices in real-world setting can enable researchers to examine this novel approach in depth and understand the measurement values.

In this study, we used commercially available Fitbit devices to gather physical activity and sleep data in adolescents. We describe our data collection and cleaning process, and present participants’ wear-time results. The collected data will be used to study associations of physical activity and sleep with many aspects of adolescent and young adult health, including cardiometabolic, experiential, and mental health, in future research.

## Methods

### Project Viva Cohort

Project Viva is an ongoing prospective cohort focused on maternal and child health. We recruited pregnant women between 1999 and 2002 in eastern Massachusetts who received prenatal care at Atrius Harvard Vanguard Medical Associates. Detailed recruitment, eligibility, and cohort information have been previously reported [26]. We collected comprehensive information from mothers and their children at various life stages, and attempted to follow all willing participants after birth. Of the 2128 mother-child pairs enrolled at birth, 1576 pairs had not previously disenrolled and thus were eligible for the Mid-Teen visit.

### Data Collection

We contacted participants from July 1, 2017, to August 30, 2021. We invited participants as mother-child pairs by mail or email to participate in the Mid-Teen visit when the child turned 16.5 years old. If the child had sibling(s) who were also enrolled in Project Viva, the sibling(s) also attended the visit regardless of the age.

We collected data on the demographic variables at several life stages. At recruitment, we collected data about the maternal education. We obtained the child sex at birth and birth date from hospital medical records. During the Mid-Teen visit, trained research assistants measured weight using a calibrated Tanita scale (model TBF-300A; Tanita Corporation of America) and weight using a calibrated stadiometer (Shorr Productions). We calculated the BMI using weight (kg)/ square of height (m^2^). Race, ethnicity, and household income were collected through a questionnaire.

### Wearable Device

We invited all adolescent participants to provide data on physical activity, heart rate, and sleep for 1 week to align with traditional actigraphy study and avoid over burdening participants. Participants had to consent to wear a Fitbit Charge 2 (before 2018) or Fitbit Charge 3 (after 2018) wearable device, depending on the year of the research visit. Both devices have the same core measurement technique, MEMS 3-axis accelerometer and optical heart-rate tracker and are able to track physical activity, heart rate, and sleep on free-living subjects [27,28]. Trained study staff instructed participants to download the Fitbit app on their smartphone and to register a Fitbit account. Study staff then linked their account ID to Fitabase [29], a data management platform to support research projects using Fitbit devices. Participants were asked to wear the device on the nondominant wrist and to synchronize their Fitbit with the app at least once daily. Once synced, study staff were able to access participant data through Fitabase. During the data collection period, research staff checked the platform 2 times a week. For those who did not provide the requested 5 days of data, we sent up to 10 reminders to participants to initiate or continue wearing the device and to sync their data on the app.

The Fitbit device measures physical activity, heart rate, and sleep stages. Physical activity is measured through miniaturized accelerometers. Fitbit uses a proprietary algorithm to calculate steps and categorizes activity intensity into 4 levels—very active, moderately active, lightly active, and sedentary, at the minute level [30,31]. The heart rate is measured through photoplethysmography, an optical technique that uses a light sensor to detect blood volume changes in the capillaries above the wrist [32]. Fitbit uses this measurement to run through a proprietary algorithm to get the beats per minute (bpm). Then, Fitbit combines the accelerometers and heart-rate pattern under a proprietary sleep algorithm to estimate sleep stages. Although some studies have implied that Fitbit may overestimate or underestimate the physical activity and sleep in certain situations, the algorithm has been validated in the contexts of measuring steps and 2-stage wake and sleep classification, with the accuracy mostly being between 80% and 90% when compared with research-grade devices [21-25,33-35].

### Data Cleaning and Analysis

We downloaded minute-level Fitbit data on August 29, 2022. The data contained information on activity intensities, steps, heart rate, and sleep every minute. We used heart rate as a proxy to determine wear time. If participants did not have heart rate observed in a given minute, we considered it as nonwear time. We used 2 cutoffs to define participants’ valid data, (1) participants with heart rate data for at least 600 minutes (10 h)/day for 5-9 days and (2) participants with heart rate data for at least 1200 minutes (20 h)/day for 5-9 days. The first cutoff is commonly used in actigraphy studies [36], while the second cutoff allows us to evaluate participants who had high compliance in a full 24-hour period [37].

We used the minute-level data to calculate the results at the daily and participant levels for valid participants. For the daily value of physical activity, we calculated the average steps/hour (during wear time), total steps per day, awake sedentary minutes, lightly active minutes, moderately active minutes, and very active minutes. The awake sedentary time was defined based on the Fitbit algorithm and excluded sleep. We then used the daily value to calculate the physical activity at the participant level.

For sleep data, we selected participants who had sleep data for 5-9 days from the 600 minutes/day cutoff described above. Fitbit assigns 3 values to indicate sleep stages for each minute during sleep periods, “1” indicates being asleep; “2” indicates being in a restless state, which may indicate restlessness during sleep or wakefulness; and “3” indicates being awake during the sleep period. Otherwise, sleep is categorized as “NA,” which indicates being fully awake (ie, not part of a sleep period) [38]. We defined a series of sleep metrics based on previous studies [39-41]. We defined sleep cycle as a series of distinct stages of sleep that a person can go through from being asleep to being awake. We defined sleep period as a specific time interval between sleep onset and the end of sleep, where multiple sleep cycles can occur in 1 sleep period. To determine the main sleep period, we manually examined participants’ sleep cycles and merged sleep cycles if multiple cycles occurred between 6 PM and 6 AM. If no sleep was found between 6 PM and 6 AM, we then examined the post 6 AM sleep onset time and manually identified the sleep period based on all available Fitbit sleep records for the participant. For the calculation of sleep duration, we focused on the main sleep period, excluding any nap times that occurred after the main sleep period.

Next, we classified the total time spent awake between the sleep onset time and sleep wake up time as wakefulness after sleep onset (WASO). We used the total asleep time between the sleep onset and wake up time divided by the total sleep duration to calculate sleep efficiency. We also calculated the sleep midpoint, which is the middle time between the sleep onset time and final wake up time. We then used the sleep midpoint to determine social jet lag, which measures the difference in sleep midpoint time between week nights (Sunday-Thursday) and weekend nights (Friday and Saturday).

We obtained the demographic characteristics for all participants who had Fitbit data available and for the subset of participants with adequate wear time based on 600 minutes/day and 1200 minutes/day cutoffs. We determined the mean (SD) for average wear days, daily wear time, total steps/hour, and total steps/day using each minimal cutoff. We then compared the wear time by sex into 4 physical activity categories: very active, moderately active, lightly active, and awake sedentary in participants who met our 600 minutes/day cutoff. Finally, we showed sleep results for all valid participants and classified participants who had average sleep onset times before and after midnight, respectively. All the data preparation and analyses were conducted using R (R Foundation for Statistical Computing).

### Ethical Considerations

The Institutional Review Board at Harvard Pilgrim Health Care approved this study protocol (235301). All participants provided written informed consent (if aged over 18 years) and assent in combination with parent or guardian informed consent (if under the age of 18 years).

## Results

### Overall

**Figure 1.**
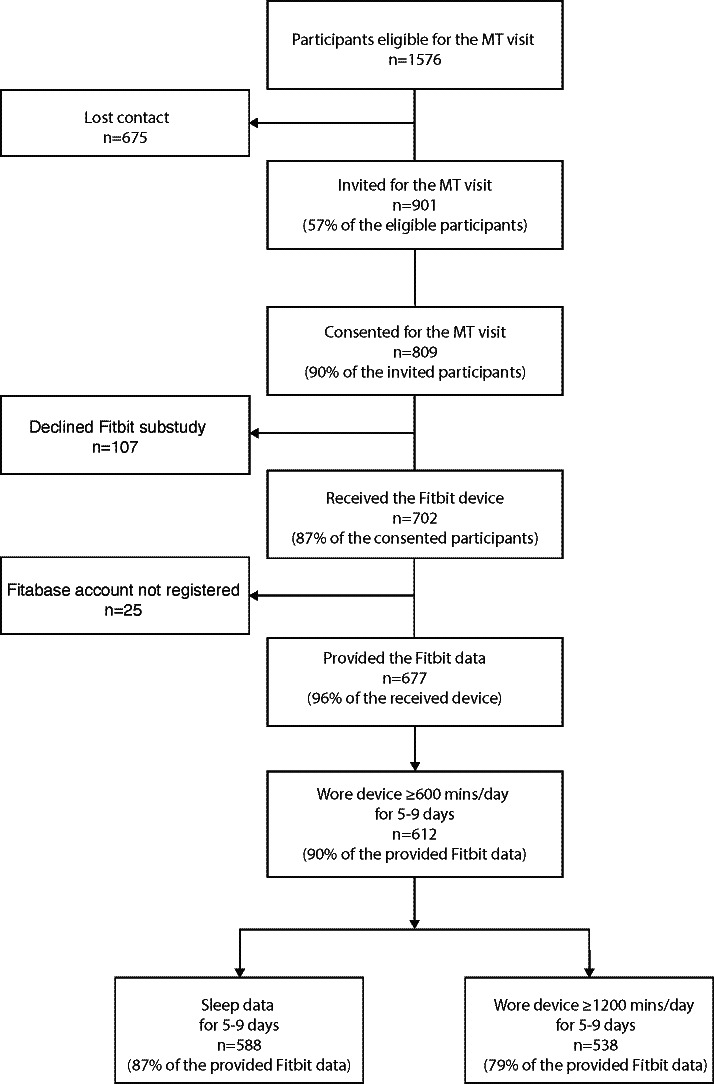
Diagram of participant flowchart showing the number of participants at different stages of the Mid-Teen (MT) visit.

Out of the 901 invited participants, 809 consented to the Mid-Teen visit and 702 agreed to participate in the Fitbit substudy (Figure 1 shows the participant eligibility flow chart). We ultimately obtained Fitbit data from 677/702 participants (96% of those who consented to the Fitbit substudy). We found similar demographic characteristics between participants who consented to the Mid-Teen visit but did not consent to the Fitbit substudy and those who consented to the Fitbit substudy (Table 1). The raw dataset comprised over 7 million minutes of physical activity and 6 million minutes of sleep data from 677 participants. After data cleaning, the percentage of valid participants remained high (612/677, 90%, using the 600 minutes/day for 5-9 days as the cutoff, and 538/677, 79%, using 1200 minutes/day for 5-9 days as the cutoff).

**Table 1.** Demographic and socioeconomic characteristics of participants who consented to the Mid-Teen visit, participants who received a Fitbit device, and participants with valid data based on 600 minutes/day and 1200 minutes/day cutoffs.

Characteristic	Participants who consented to the Mid-Teen visit (n=809)	Participants who consented to the Mid-Teen visit but did not provide Fitbit data (n=132)	Participants who received the Fitbit device (n=677)	Participants with valid data considering the cutoff of 600 minutes/day for 5‐9 days (n=612)	Participants with valid data considering the cutoff of 1200 minutes/day for 5‐9 days (n=538)
Age (years), mean (SD)	17.4 (0.7)	18.3 (0.8)	17.7 (0.7)	17.7 (0.7)	17.7 (0.7)
Sex at birth, n (%)					
Female	423 (52)	67 (51)	356 (53)	332 (54)	295 (55)
Male	386 (48)	65 (49)	321 (47)	280 (46)	243 (45)
Race and ethnicity, n (%)					
Non-Hispanic White	528 (65)	89 (67)	439 (65)	395 (65)	346 (64)
Non-Hispanic Black	121 (15)	27 (20)	94 (14)	85 (14)	76 (14)
Hispanic	78 (9.6)	9 (6.8)	69 (10)	60 (9.8)	50 (9.3)
Non-Hispanic Asian	26 (3.2)	4 (3)	22 (3.2)	22 (3.6)	22 (4.1)
Other	56 (6.9)	3 (2.3)	53 (7.8)	50 (8.2)	44 (8.2)
BMI (kg/m^2^), mean (SD)	23.96 (5.2)	22.65 (2.8)	24.01 (5.3)	23.94 (5.2)	24.03 (5.3)
Unknown, n (%)	114 (14.1)	104 (79)	10 (1.5)	9 (1.5)	9 (1.7)
Household income, n (%)					
<$40,000	51 (6.3)	10 (7.6)	41 (6.1)	34 (5.6)	33 (6.1)
$40,000-$70,000	79 (9.8)	15 (11)	64 (9.5)	54 (8.8)	45 (8.4)
>$70,000	597 (74)	72 (55)	525 (78)	482 (79)	423 (79)
Unknown	82 (10)	35 (27)	47 (6.9)	42 (6.9)	37 (6.9)
Maternal college education, n (%)	598 (74)	103 (78)	495 (73)	453 (74)	400 (75)

### Participant Compliance

Among all participants with valid data considering the cutoff of at least 600 minutes/day (on every day worn), participants tended to wear the device for more than 10 hours per day and for more days than requested (Figure S1 in Multimedia Appendix 1). Furthermore, the pattern of wearing the device for more than 10 hours per day persisted when comparing the first recording day with the last recording day (Table S1 in Multimedia Appendix 1). When comparing the wear time between the two cutoffs, the average number of wear days and daily wear time were similar between the 600 minutes/day cutoff group and the 1200 minutes/day cutoff group (wear days: 7.5 days, SD 1.1 days vs 7.7 days, SD 0.9 days; wear time: 1323 minutes, SD 100 minutes vs 1350 minutes, SD 61 minutes). Similarly, the average values for steps/hour were similar between the groups defined by the two different cutoffs (Table 2).

**Table 2.** Participants’ physical activity results based on 600 minutes/day and 1200 minutes/day cutoffs.

Variable	Participants with valid data considering the cutoff of 600 min/d wear time for 5‐9 days (n=612), mean (SD)	Participants with valid data considering the cutoff of 1200 min/d wear time for 5‐9 days (n=538), mean (SD)
Total wear time (d)	7.5 (1.1)	7.7 (0.9)
Daily wear time (min)	1323 (100)	1350 (61)
Total steps per hour (steps/h)	399 (157)	400 (150)
Total steps per day (steps)	8883 (3455)	9041 (3414)
Physical activity level		
Very active (min/d)	20 (20)	21 (20)
Moderately active (min/d)	23 (18)	23 (18)
Lightly active (min/d)	235 (70)	239 (67)
Awake sedentary (min/d)	564 (138)	568 (121)

### Physical Activity Results

**Figure 2.**
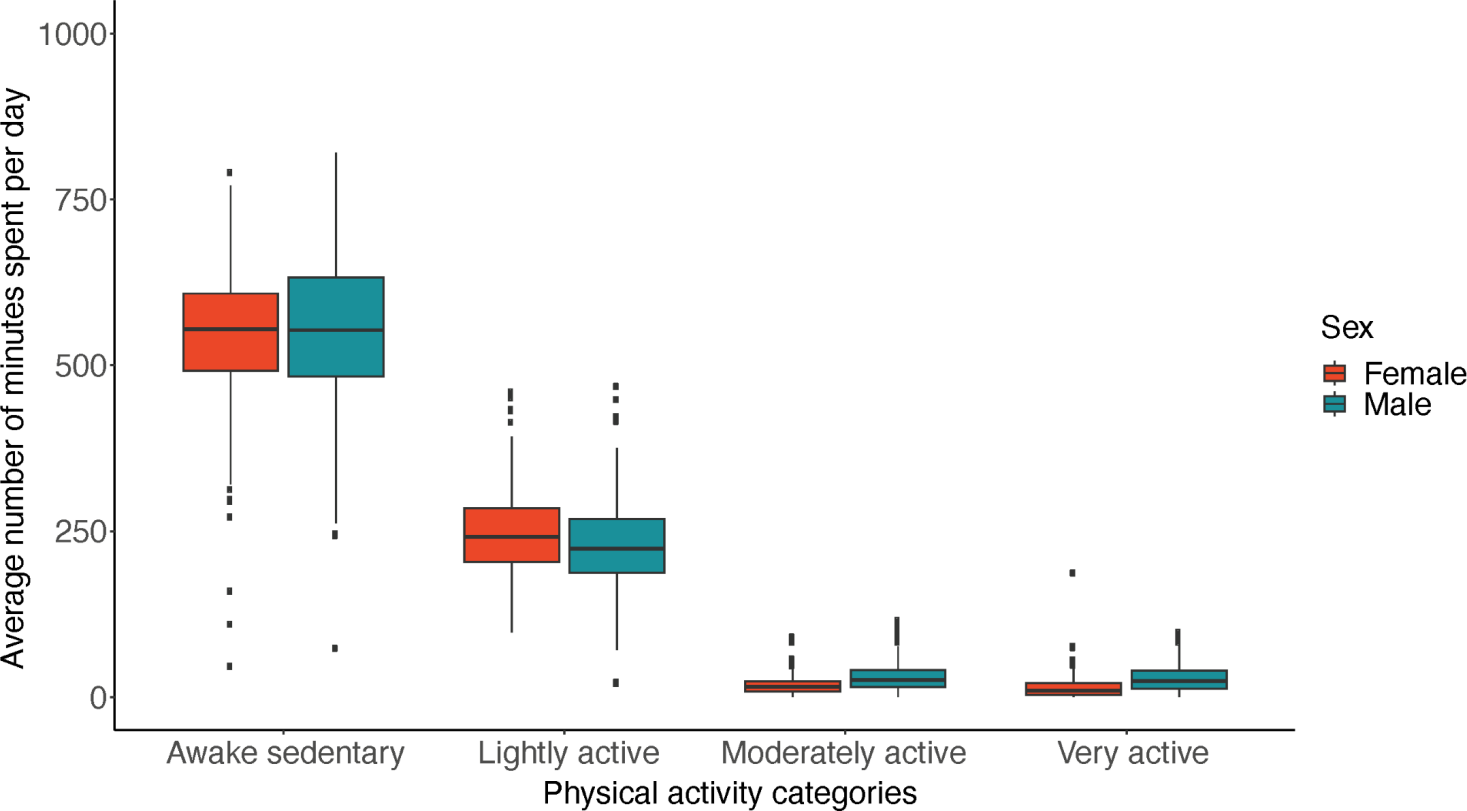
Average time spent per day in each of the 4 activity levels: awake sedentary, lightly active, moderately active, and very active. Orange represents female participants and teal represents male participants. The vertical axis indicates the wearing time spent in each activity. Participants were selected using the minimal cutoff for wear time of at least 600 minutes/day for 5‐9 days.

Among the 4 physical activity categories (awake sedentary, lightly active, moderately active, and very active), we found that of the total awake wear time, the longest duration was spent in sedentary activity for both the 600 minutes/day cutoff group (mean 564 min/d, SD 138 min/d) and the 1200 min/d cutoff group (mean 568 min/d, SD 121 min/d; Table 2). The average wear time spent in the physical activity categories of very active and moderately active was less than 25 minutes per day for both cutoffs. Male participants spent a slightly higher wear time in the physical activity categories of very active and moderately active compared with female participants (very active: 27 min/d vs 15 min/d, moderately active: 29 min/d vs 17 min/d; Figure 2). On the other hand, female participants spent slightly more time in light activity compared with male participants (245 min/d vs 222 min/d).

### Sleep Results

**Figure 3.**
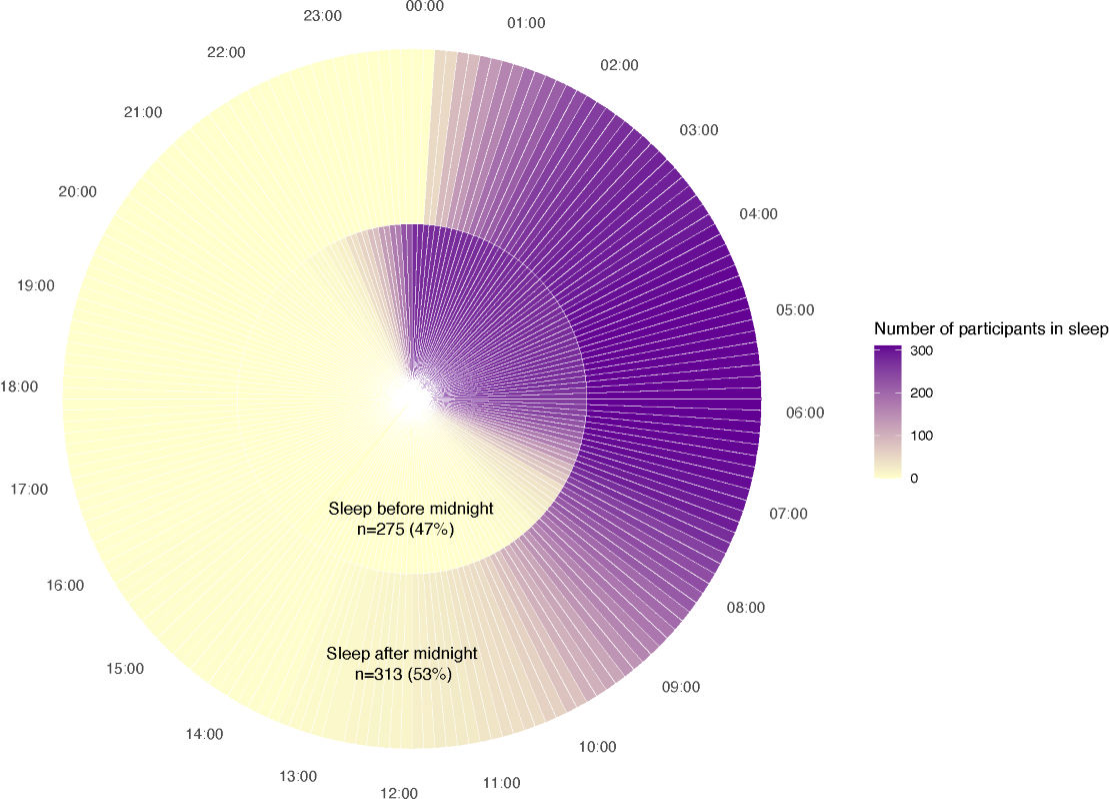
Sleep patterns for participants with average sleep onset before or after midnight (N=588). The inner circle includes participants whose average sleep onset time occurred before midnight (275/588, 47%) and the outer circle includes participants whose average sleep onset time occurred after midnight (313/588, 53%). The clock is based on 24-hour clock with 15 minute intervals. The gradient represents the number of participants in sleep in that given interval, where more purple color indicates more participants in sleep.

Out of the 612 participants who wore the Fitbit for at least 600 minutes/day, 588 participants had sleep data available (Table 3). In these participants, the average number of days with sleep data available was 6.9 (SD 1.2) days with a mean sleep duration of 7.9 (SD 0.9) hours and average sleep onset time of 00:16 AM (SD 1.5 h). When stratified by weekends and weekdays, participants exhibited longer sleep duration during weekends compared with weekdays (mean 8.4 hours, SD 1.4 hours vs 7.8 hours, SD 1.1 hours), while the sleep efficiency and WASO stayed consistent (Table S2 in Multimedia Appendix 1). In addition, we classified participants into 2 groups (average sleep onset time before midnight vs average sleep onset time after midnight). The sleep efficiency and WASO were minimally different between the two groups. However, the group with the average sleep onset time before midnight had a longer sleep duration, more social jet lag, and earlier wake up time compared with the second group (sleep duration: 8.1 hours, SD 0.8 hours vs 7.8 hours, SD 0.95 hours; social jet lag: 1.0 hour, SD 1.2 hours vs 0.6 hours, SD 1.9 hours; and wakeup time: 07:16 AM, SD 0.9 h vs 09:09 AM, SD 1.6 h). Furthermore, Figure 3 shows that participants with sleep onset after midnight have a wider wake window (a larger SD) compared with participants with sleep onset before midnight.

**Table 3.** Sleep results for participants who have valid data for 5‐9 days of sleep considering 600 minutes/day, and separated by sleep onset before midnight and sleep onset after midnight.

Sleep measure	Overall participants (n=588), mean (SD)	Participants with average sleep onset before midnight(n=275), mean (SD)	Participants with average sleep onset after midnight(n=313), mean (SD)
Number of days with available data	6.9 (1.2)	6.9 (1.3)	6.9 (1.2)
Sleep duration (h)	7.9 (0.9)	8.1 (0.8)	7.8 (0.95)
Sleep efficiency (%)	93 (5)	93 (6)	93 (5)
Wakefulness after sleep onset (h)	0.6 (0.4)	0.6 (0.5)	0.6 (0.4)
Social jet lag (h)	0.8 (1.6)	1.0 (1.2)	0.6 (1.9)
Sleep onset time	00:16 AM (1.5 h)	23:11 PM (0.6 h)	01:17 AM (1.2 h)
Wake-up time	08:17 AM (1.6 h)	07:17 AM (0.9 h)	09:09 AM (1.6 h)

## Discussion

### Principal Findings

Physical activity and sleep are major behavioral factors associated with various domains of health in adolescents. Consumer-based accelerometry devices, such as Fitbit, have enabled researchers to examine these behaviors efficiently at scale. For our Mid-Teen visit (aged ~17 years old), we invited Project Viva participants to use a Fitbit wearable device to collect physical activity and sleep data. We found participants were likely to consent and to wear the devices for the requested time. We observed that adolescents were spending the majority of their awake time in sedentary activities, with very little time participating in the physical activity categories of very active and moderately active. In the sleep data, we noticed that sleep onset time minimally impacted average sleep duration, sleep efficiency, and WASO; however, participants with a sleep onset after midnight tended to have much wider wakeup time window and less social jet lag.

The rising popularity of wearable devices has introduced a new method of data collection for assessing physical activity and sleep for epidemiology research [42]. This study illustrated the feasibility of using consumer-based wearable devices to collect human-behavior data, such as physical activity, sleep duration, and other sleep metrics, in free-living conditions. In addition, the high granularity of this data enabled us to examine physical activity and sleep at multiple levels: minute level, daily level, and the participant level. The objective data collection method avoided measurement errors resulting from recall or social desirability bias [43,44]. The device being used, unlike many research-based devices, does not need to be mailed back by participants, which avoided additional communication and shipping costs for the researchers, and participant burden of shipping back devices. We additionally avoided the potential of device and data loss during shipping. Furthermore, in the initial communications for the Mid-Teen visit, study staff informed participants that they could keep the device after the study period, and many participants felt it was a nice incentive to participate.

Project Viva has collected sleep and cardiometabolic health information across multiple visits in childhood and adolescence, and plans to continue health assessments in many domains in young adulthood. Our physical activity findings align with existing research, emphasizing the prevalence of sedentary lifestyle among adolescents [45,46]. Notably, we observed a delayed sleep onsite time and longer sleep duration on weekends, similar to previous studies [47,48]. For our future studies, we plan to examine associations of sleep and physical activity behaviors with cardiometabolic health data already collected at the midadolescence visit, such as weight, body composition, blood pressure, and blood biomarkers of cardiovascular health. Furthermore, we plan to use the Fitbit results as a calibration tool to enhance the accuracy and reliability of the self-reported physical activity and sleep data for participants who did not participate in the Fitbit substudy. These Fitbit data offer a unique way to examine health behavior and provide valuable insights into the relationship between behavioral factors and chronic disease development with opportunities for potential interventions. In addition, we have minute level and daily level dataset on objective health behaviors available for more detailed analyses.

### Limitations and Challenges

While this study provides insights in using wearable devices to collect adolescent behavior data, there are some limitations to the inferences we can make. First, unlike other actigraphy studies that require participants to complete sleep diaries to self-report sleep onset and wake up times while wearing the device, our study did not request participants to fill out sleep diaries. This made it more difficult to clean the sleep data, especially for participants whose sleep period was less consistent, requiring us to make judgments based on their recorded sleep patterns and manually assign “sleep periods.” Second, despite instructing participants to wear the device on the nondominant wrist, we were unable to actively track the wearing status of the device. The wear habits might have influenced the accuracy of step counts and sleep stage recording [49-51]. The third limitation is the proprietary algorithm used by Fitbit. Although previous validation studies have shown 80%‐90% of accuracy for Fitbit devices in adolescents and adults when comparing both physical activity and sleep using research-grade accelerometry[21-25,50-52], the lack of accessibility to Fitbit’s underlying algorithms poses challenges in understanding the logic behind the algorithms for each device type and age populations. Finally, Project Viva is composed of adolescents from families with generally high median incomes and higher educational background, which could restrict the ability to generalize the findings to other populations. However, the high granularity data captures individual differences and can be used for comparative analyses as well as integrating with other cohort to extend the findings to a diverse population.

### Conclusion

This study provided valuable insights into using consumer-based wearable devices to collect human behavior data. These data on physical activity and sleep characteristics are important for researchers seeking to understand their influence on chronic disease development and mental health outcomes. For future research, consumer wearable devices hold great potential for researchers to apply across different adolescent populations. Their use allows us to gain greater understanding of how lifestyle factors impact long-term health outcomes in diverse populations. These data may shed light on future policies or interventions aiming at increasing physical activity and improving sleep health, ultimately leading to improvements in physical and mental health.

## Supplementary material

10.2196/59159 Multimedia Appendix 1 Basic statistical results.

## Abbreviation

BPM: beats per minute
WASO: wakefulness after sleep onset
