# Supplementary material for: Using a Consumer Wearable Activity Monitoring Device to Study Physical Activity and Sleep Among Adolescents in Project Viva: Cohort Study
Source: JMIR Pediatr Parent. 2025 Feb 4;8:e59159. doi: 10.2196/59159 (PMC11813160; doi:10.2196/59159)
Supplement: Multimedia Appendix 1 [file pediatrics-v8-e59159-s001.docx]

**Figure S1.** Fitbit results overview at participant-level: distribution of participants’ total wear days, daily wear time, total steps per day and total steps per hour. Results were based on a wear time cutoff of at least 600mins/day, N=612 participants

**Table S1.** Participants wear time and total steps per hour in the first recording day and last recording day.

|  | First recording day (N=612)  Mean (SD) | Last recording day (N=612)  Mean (SD) |
| --- | --- | --- |
| Wear time (minutes) | 1222 (294) | 1292 (217) |
| Total Steps per hour (steps/hour) | 412 (249) | 380 (241) |

**Table S2.** Sleep results for participants who have 5 -9 days of sleep data in 600 minutes/day valid participants, separated by weekends and weekdays

|  | Participants with average sleep onset during weekdays  (N=588) Mean (SD) | Participants with average sleep onset during weekends  (N=581) Mean (SD) |
| --- | --- | --- |
| Available days | 4.8 (1.0) | 2 (0.5) |
| Sleep Duration (hours) | 7.8 (1.1) | 8.4 (1.4) |
| Sleep efficiency (%) | 93 (6) | 93 (6) |
| Wakefulness After Sleep Onset (hours) | 0.6 (0.4) | 0.6 (0.5) |
| Sleep onset time | 00:10 AM  (1.5 hours) | 00:40 PM  (1.6 hours) |
| Wake-up time | 07:56 AM  (1.8 hours) | 09:08 AM  (1.8 hours) |
